# Editorial on research topic: Surgery and COVID-19 in oncologic patients: What does the recent coronavirus pandemic taught us?

**DOI:** 10.3389/fsurg.2022.1081959

**Published:** 2023-01-10

**Authors:** Ugo Cioffi, Marco Chiarelli, Alberto Testori, Matilde De Simone, Michele M. Ciulla, Matteo Calderoni, Enrico Cassina, Marco Scarci, Federico Raveglia

**Affiliations:** ^1^ Department of Surgery, University of Milan, Milano, Italy; ^2^ Emergency and Robotic Surgery, A. Manzoni Hospital, ASST Lecco, Lecco, Italy; ^3^ Department of General and Thoracic Surgery, Humanitas Research Hospital, Rozzano, Italy; ^4^ Laboratory of Clinical Informatics and Cardiovascular Imaging, Department of Clinical Sciences and Community Health, University of Milan, Milan, Italy; ^5^ Scuola di Specializzazione Chirurgia Toracica, Università Degli Studi di Milano, Milano, Italy; ^6^ Thoracic Surgery, ASST Monza, Monza, Italy; ^7^ Department of Thoracic Surgery, Imperial College Healthcare NHS Trust, London, United Kingdom

**Keywords:** COVID - 19, surgery, oncologic patient, pandemic, strategy, infection

**Editorial on the Research Topic** Surgery and COVID-19 in oncologic patients: What does the recent coronavirus pandemic taught us?

The Coronavirus outbreak has recently shocked the world and the health systems as well, overwhelming most hospitals and departments. The need to give universal recommendations has become mandatory during this health emergency, however many countries have reacted without an agreement or consensus flow-charts on the strategies to be adopted. This helped the infection spread into a pandemic given Sars-Cov-2 high transmission rate. Two main critical issues seemed to hit hard most of the health systems: the number of healthcare personnel who contracted the infection and the availability of hospital beds, mainly in ICU. The need for beds for COVID-19 patients and the lack of medical staff has severely impacted on surgical departments causing the delay or even the cancellation of many operations during the different waves of the pandemic, including in the oncologic field.

To overcome this challenging point a change of mind was mandatory. Therefore, we designed a research topic in which the latest suggestions and strategies on how to deal with the oncological patient where collected, by the use of an alternative way of managing the waiting list for surgery. Several Authors have addressed this issue from a 360° point of view, ranging from biology to management, focusing in particular on:

• the impact of Covid-19 on tumor behavior;
• the effect of the delayed surgery on perioperative and long-term outcomes for most common tumors;
• optimal patients triage based on tumor stage;
• alternative therapeutic treatments involving oncologists and radiotherapists;
• optimization of patients’ pathway during the hospital stay to prevent any contamination;
• resources redistribution.

Although International Societies such as the Society of Surgical Oncology have published guidelines for non-emergency surgical procedures, the effect of the acceptable waiting time between the diagnosis and surgery has not yet been well defined and a possibility of a worsened clinical outcome is still unclear. It is known that due to immunosuppressive responses and pro-inflammatory cytokines release, surgery could lead to a high risk of COVID-19 during hospital stay (1).

The authors evaluated the impact of the pandemic on the reorganization of the surgical wards, the effect of delayed surgery for outcomes like overall survival, tumor burden, and the number and grade of complications. They also went to analyze the effects of the Coronavirus infection of the tumor microenvironment and its influence on the spread of premetastatic cells.

Therefore, Jichun et al. in their review evaluated the impact of the delay of gastrectomy in gastric cancer patients’ survival. Their research showed that postponing until 8 weeks surgeries for patients with a diagnosis of gastric cancer did not affect outcomes such as OS and DFS.

In the spasmodic quest to obtain the best results from surgery in cancer patients, many researches have been done to indicate the right therapeutic window. Based on the type of tumor, many studies increasingly conclude that a shortening of the waiting period seems to be beneficial in terms of main outcomes and tumor spread. In a historical period like this, however, trying to get the best out of the limited resources available can be complex and this article shows us how a slight delay in treatment can be considered acceptable, while guaranteeing excellent results for patients (2).

In their manuscript Ruixian et al. analyzed how the various phases of the pandemic characterized a delay in the treatment of breast cancer patients. Comparing the data, they showed that the phases between the pandemic peak at the beginning of 2020 and the post-peak phase and the plateau phase of the infectious curve resulted in different timing for surgery (TTS). Patients with diagnostic breast cancer at different times of the pandemic were therefore forced to increase waiting times, as expected, in the peak phase and shorter delays in the plateau phase.

In their research Carissimi and the surgical team of the S. Gerardo Hospital retrospectively analyzed data of their Hepato-Bilio-Pancreatic (HBP) mid-volume surgery center to look for any differences caused by the reorganization of the hospital system. In terms of length of hospital stay, morbidity and mortality, there were no differences between the groups of patients operated on in the period prior to the pandemic and during that. This was possible thanks to a careful separation of departments able to accommodate negative Covid patients and to keep the ward as such thanks to serial controls with protocol of testing spread all over the world. Despite the need to provide health personnel to cover shifts in wards of patients affected by Covid-19, the Monza Hospital has been able to remain a third-level center for oncological HPB pathology, thus creating safe departments for patients. However, the analysis inevitably showed an increase in tumor burden in patients operated on during the pandemic period, demonstrating a significant effect of the delay between the period of diagnosis, the surgical indication and the operation itself. In the same way, the follow-up and checkup times have obviously increased. Therefore, the significance of ordering clear separation to create safe pathways for these patients in need of surgery seems to be consistent.

As shown in the manuscript by Xiahoao Zheng et al. the prolongation of the waiting time does not appear to have adversely affected the number and severity of short-term complications of patients treated with total gastrectomy in the National Cancer Center. Fragile patients such as those undergoing total gastrectomy could benefit from early surgery given the advanced degree of the disease, although an increase in waiting time does not appear to have affected short-term complications.

Dr. Aramini et al. instead went to study the effect of Covid-19 infection on the tumor microenvironment in patients affected by the virus. As is now known, the Coronavirus infection prefers in its most severe forms an involvement of the cardio-pulmonary district with consequent multi-organ failure. The colleagues’ study analyzed the effects of activating the macrophage-neutrophil cascade at the level of the tumor microenvironment in lung cancer. Many studies have already shown how a pro-cytokine cascade can influence a reactivation of the immune system at the level of tumor foci with consequent spreading of micrometastases at a local level and the activation of extracellular neutrophil traps has been shown to create a spread of premetastatic cells in the lung. The deeper analysis of these phenomena will help in the development of further targeted therapies, both for cancer prevention and for the treatment of patients with COVID-19 (3).

To conclude, the management of cancer patients in a pandemic context can be very challenging but thanks to the amount of data collected in the last 2 years, new strategies can be adopted to make the best use of resources. The road travelled during this pandemic has certainly put us to the test, but the efforts made seem to have paid off to get out of the dark period. First, a reorganization and the establishment of Covid-free hubs has been a successful strategy. As Anna M. Perrone and colleagues at the University of Bologna Hospital have shown, prioritization of oncological surgical care and the allocation of resources during a pandemic in COVID-19 free surgical hubs is an appropriate choice to guarantee oncological protocols and to obtain high-standard results. It must also be admitted that the pandemic has unmasked the weak side of many Health Systems, exposing gaps already present in the years. However, the pandemic and its teachings could represent the opportunity for an advantageous restart at the same time (4–6).

A more careful organization and an effective and wise use of resources will certainly guarantee us a better treatment for this class of fragile patients and will allow us to insert more precise attitudes into clinical practice and as the main coauthor of COVID-Surg Collaborative stated, “guaranteeing the possibility of safely carrying out elective cancer surgery should be part of the health plans of each country to protect the health of the entire population” (7). Indeed, “when it is obvious that goals cannot be reached, don’t adjust the goals, adjust the action steps”.

## References

[1] Cavaliere D Parini D Marano L Cipriani F Di Marzo F Macrì A Surgical management of oncologic patient during and after the COVID-19 outbreak: practical recommendations from the Italian society of surgical oncology. Updates Surg. (2021) 73:321–9. 10.1007/s13304-020-00921-4 33184782 PMC7660129

[2] Nilssen Y Brustugun OT Tandberg Eriksen M Gulbrandsen J Skaaheim Haug E Naume B Decreasing waiting time for treatment before and during implementation of cancer patient pathways in Norway. Cancer Epidemiol. (2019) 61:59–69. 10.1016/j.canep.2019.05.004 31153048

[3] Turnquist C Ryan BM Horikawa I Harris BT Harris CC. Cytokine storms in cancer and COVID-19. Cancer Cell. (2020) 38(5):598–601. 10.1016/j.ccell.2020.09.019 33038939 PMC7531591

[4] Cioffi U Ciulla MM De Simone M Scarci M Testori A Raveglia F Editorial: surgery and COVID-19: which strategies to apply in oncologic patients. Front Surg. (2021) 8:718751. 10.3389/fsurg.2021.718751 34368220 PMC8339367

[5] Raveglia F Orlandi R Li H Cerfolio R Tam JKC Scarci M. Editorial: rethink thoracic surgery as a whole after the pandemic. How to optimize resources and deliver excellent patient care. Front Surg. (2022) 9:920626. 10.3389/fsurg.2022.920626 35706849 PMC9191574

[6] Raveglia F Orlandi R Rimessi A Minervini F Cioffi U De Simone M Standardization of procedures to contain cost and reduce variability of care after the pandemic. Front Surg. (2021) 8:695341. 10.3389/fsurg.2021.695341 34250010 PMC8264450

[7] COVIDSurg Collaborative. Effect of COVID-19 pandemic lockdowns on planned cancer surgery for 15 tumour types in 61 countries: an international, prospective, cohort study. Lancet Oncol. (2021) 22(11):1507–17. 10.1016/S1470-2045(21)00493-9 34624250 PMC8492020

